# Analysis of age, period, and birth cohort effects on suicide mortality in Brazil and the five major geographic regions

**DOI:** 10.1186/s12889-023-16289-0

**Published:** 2023-07-13

**Authors:** Pauliana Valéria Machado Galvão, Cosme Marcelo Furtado Passos da Silva

**Affiliations:** 1 School of Medicine, Serra Talhada Campus, University of Pernambuco, Pernambuco, Brazil; 2 Department of Epidemiology and Quantitative Methods in Health, National School of Public Health Sergio Arouca, Oswaldo Cruz Foundation, Pernambuco, Brazil

**Keywords:** Mortality, Cohort effect, Period effect, Age effect, Brazil, Age-period-cohort analysis

## Abstract

**Objective:**

Estimate the effects of age, period, and birth cohort on suicide mortality in Brazil by major geographic region in the overall population and by sex.

**Methods:**

This was a time trend ecological study. National and regional suicide mortality data from 1981 to 2020 were analyzed for the overall population and by sex. Age, period, and cohort effects were calculated with a Poisson regression model using estimable functions with the Epi package of the R statistical program, version 4.2.1.

**Results:**

There were 272,716 suicides in individuals ranging from 20 to 79 years old. In the overall population, the age model-adjusted suicide mortality rates showed an upward pattern for Brazil. The most recent cohort showed the highest associated risk, 1.67 (95%CI 1.63; 1.71), while for the reference period, it was the highest risk among all the periods.

**Conclusions:**

Suicide mortality rates have shown an upward trend with advancing age in both men and women in the Brazilian population. However, the behavior of the period effect and cohort depends on the population analyzed and regional distribution.

## Introduction

Dealing with death refers to fears, uncertainties, and feelings of loss and grief, which increase exponentially regarding suicide [1]. It can be considered a conscious act of self-annihilation experienced by the one in a situation of vulnerability, who perceives it as the best solution to get out of an unbearable psychological pain [2, 3]. It results from a complex biological, genetic, psychological, social, cultural, and environmental interaction. It is difficult to explain why some people commit suicide while others, in a similar or worse situation, do not [4].

According to the World Health Organization (WHO), 703 thousand people die due to suicide per year and many more who attempt [5]. Even though the highest suicide rates are concentrated in countries in Asia and Europe, Brazil has one of the highest rates in absolute numbers worldwide [6, 7]. For years, Brazil reported lower suicide rates than other countries [8].

The background for this phenomenon is strongly influenced by the accelerated epidemiological transition process [9]. The Brazilian population underwent various changes starting in the latter half of the 20th century, launching a demographic transition. First, infant mortality declined, life expectancy increased, and birth and fertility rates were still high. In the mid-1960s, the latter rates began to drop, which intensified in the 1970s. Since then, the impact of infectious and parasitic diseases has decreased among the causes of illness and death in the Brazilian population, while external causes and chronic noncommunicable diseases have taken more victims [10]. In the wake of this phenomenon, the last five decades have witnessed a significant increase (60%) in Brazil’s suicide rates [11, 12]. Between 2001 and 2019, these rates ranged from 5.3 to 6.6 deaths per 100,000 inhabitants, reaching 13,523 deaths in 2019 [13, 14].

As in other countries, a gender difference is notable. The highest frequency is among men, at an approximate male: female ratio of 4/1 [12, 15–17]. This difference between men and women is often attributed to more lethal suicide attempt methods, greater aggression, and higher intent to die among men [18].

Another issue in a country of continental dimensions such as Brazil is the regional differences found. Higher rates have been found in the South, Southeast, Central-East [19], Northeast [19–21], and North [19, 21, 22], plus the highest percentage growth has been in the Northeast and Central-East [23, 24].

Furthermore, changes in suicide rates and the age profile of individuals who commit suicide have raised new research questions. Preliminary studies have shown directly or indirectly the effects of birth cohort, that is, generational differences in suicide risk [25]. A key objective in epidemiology is to identify influences on different factors for illness and death over time [26], and one approach for attempting to understand suicide behavior in society has been age-period-cohort (APC) modeling [25, 27–33]. This modeling provides a descriptive tool for observing disease records and the temporal effect of an event’s occurrence [34–36], highly useful for modeling incidence and mortality rates [37].

APC analysis has the unique capacity to moderately describe the entire complex of social, historical, and environmental factors that simultaneously affect individuals and populations of individuals and is widely used to address questions of lasting importance for studies on social change, disease etiology, aging, and population processes and dynamics [34]. APC modeling serves to separate the temporal effects of age, historical circumstances (period), and generational succession (birth cohort) [25, 34]. However, the modeling could be improved by the redundancy of linear effects of age, period, and cohort since any two dimensions fix the third, a problem called identifiability, a widely debated fact [25, 37].

This study aimed to estimate the effect of age, period, and birth cohort on suicide rates in Brazil and its five major geographic regions, using the APC approach, in the overall population and by sex.

## Materials and methods

This was an ecological study on suicide mortality in Brazil from 1981 to 2020. Data were obtained from the death certificates recorded in the Mortality Information System (SIM), and the population estimates were from the Brazilian Institute of Geography and Statistics (IBGE), downloaded from the website of the IT Department of the Unified Health System (DATASUS).

There were 307,740 suicides recorded during the study period. The current study included suicide deaths in individuals between 20 and 80 years (due to the need for age brackets with regular intervals). Thus, 35,024 suicides (11,3%) were removed from the sample, making 272,716 suicides eligible for the proposed modeling.

Of the excluded records, 702 (0.2%) had no information on age and date of birth, 6,289 (2.0%) corresponded to individuals 80 years of age or older, and 28,033 (9.1%) corresponded to individuals under 20 years of age. The records with ignored age were excluded because they did not allow the use of the proposed modeling, while the records of individuals aged 80 years or older were excluded because there are no population records that group the data into quinquennia (80 to 84 years, 85 to 89 years, …). Finally, the group of individuals under 20 years of age presents disagreements regarding the initial age of perception of death and intentionality of death, While there are researchers who argue that children under the age of 10 are rarely able to understand the finality of death and therefore do not understand the intentionality of death and suicide [38], there are reports of early onset of suicidal behaviors in 4-5-year-olds, but there is no consensus of the understanding of the process at this age [18, 39, 40]. Furthermore, analyzing suicide in adolescents in a comparative profile with other age groups may minimize the importance of this grievance since the rates are proportionally much lower than in age groups over 20 [39].

The data were compiled in the R software, version 4.2.1, and the modeling was done with the Epi package, ggplot2, and gridExtra.

Death records were initially corrected for information quality through proportional redistribution of deaths due to Events of Undetermined Classification (ECI), according to year, age group, and region, based on four steps [41]: 1) the proportion of deaths due to suicide was calculated concerning the total number of deaths due to external causes; (2) the value obtained in the previous step was multiplied by the total number of deaths classified as ECI; (3) the result of the second stage was added to the total number of deaths initially classified as suicide, representing the corrected registration of these deaths; (4) the coverage of deaths was rectified by correction factors based on Queiroz et al. [42], Lima e Queiroz [43] e Silva [44]. This correction is necessary to avoid the influence of poor data quality on time-series mortality investigations [45, 46].

With the corrected data, crude and adjusted mortality rates were calculated for each year, and the adjusted rates calculated by the direct method and using the standard population recommended by the WHO [47], to allow comparison of the rates by year.

For the age-period-cohort (APC) modeling, the number of deaths and the population at risk were grouped into 12 age brackets (20–24 years; 25–29 years; 30–34 years; 35–39 years; 40–44 years; 45–49 years; 50–54 years; 55–59 years; 60–64 years; 65–69 years; 70–74 years; and 75–79 years), for 7 periods (1981–1985; 1986–1990; 1991–1995; 1996–2000; 2001–2005; 2006–2010; 2011–2015; 2016–2020) and 18 cohorts (1906–1910; 1911–1915; 1916–1920; 1921–1925; 1926–1930; 1931–1935; 1936–1940; 1941–1945; 1946–1950; 1951–1955; 1956–1960; 1961–1965; 1966–1970; 1971–1975; 1976–1980; 1981–1985; 1986–1990; 1991–1995; 1996–2000). The cohorts were not furnished, since the selected function calculates the cohort based on period and age.

Age, period, and cohort effects were modeled with natural splines for each of the terms and calculated via Poisson regression, expressed as:

$$\text{ln}\left(E\left[{r}_{ij}\right]\right)= \text{ln}\left(\frac{{\theta }_{ij}}{{N}_{ij}}\right)=\mu +{\alpha }_{i}+{\beta }_{j}+{\gamma }_{k}$$

where the logarithm of the rate’s expected values is a linear function of the effect of age, period, and cohort; $E\left[{r}_{ij}\right]$ represents the expected mortality rate at age *i* and period *j*; ${\theta }_{ij}$and ${N}_{ij}$ represent the number of deaths and the population at risk, respectively, at age *i* and in period *j*; *µ* represents the mean effect; *α*_*i*_, the effect of age *i*; *β*_*j*_, the effect of period *j*; and *γ*_*k*_, the effect of cohort *k* [45, 48–51].

Compared submodels were presented in the results session in nested form by age, age-drift^a^, age-cohort, age-period-cohort, age-drift^b^, the superscripts a and b being the cohort drift and period drift models, respectively. The comparison of the models in Table 1 is made by the likelihood ratio test between two sub-models, namely: (1) compares the age and age-drift models: if significant represents a non-linear effect of age; (2) compares the age-drift and age-cohort models: if significant represents a non-linear effect of cohort; (3) compares the age-cohort and age-period-cohort models: if significant represents nonlinear effect of period, in the presence of cohort; (4) compares the age-period-cohort and age-period models: if significant represents nonlinear effect of cohort, in the presence of period; (5) compares the age-period and age-drift models: if significant represents nonlinear effect of period [34].

**Table 1:** Suicide mortality rates by age group and period. Brazil and Regions. 1981 to 2015

Age Group	Cohort							
	1906–1910	1986–1990	1991–1995	1996–2000	2001–2005	2006–2010	2011–2015	2016–2020
**Brasil**								
20–24 years	27.24	23.94	27.27	28.82	29.82	30.20	30.85	36.86
25–29 years	27.34	25.21	28.85	29.59	31.52	31.54	33.81	36.86
30–34 years	26.64	25.80	29.48	29.57	31.08	32.32	34.70	36.61
35–39 years	26.06	25.62	29.97	33.01	32.41	34.32	35.90	38.38
40–44 years	27.21	24.85	29.73	33.33	36.16	36.97	37.26	39.35
45–49 years	29.77	29.59	32.79	35.14	38.31	37.35	37.50	41.38
50–54 years	30.72	28.61	33.37	36.53	38.81	35.83	37.56	41.88
55–59 years	32.47	31.83	33.26	36.43	37.80	35.81	37.41	41.20
60–64 years	30.29	31.78	31.50	35.06	36.08	35.31	35.64	39.79
65–69 years	31.56	31.17	35.46	36.94	34.91	35.59	37.65	41.87
70–74 years	34.77	39.84	40.50	38.16	37.39	35.44	40.78	43.89
75–79 years	38.85	39.88	41.57	43.52	39.66	35.62	40.20	44.77
**North**								
20–24 years	37.68	33.64	39.71	37.08	43.09	40.66	45.18	51.55
25–29 years	28.96	34.72	37.79	30.93	37.05	34.94	43.63	41.38
30–34 years	27.35	25.49	26.41	28.47	33.12	32.74	38.09	38.48
35–39 years	28.13	29.34	33.89	28.66	30.18	30.24	36.03	35.84
40–44 years	27.67	23.22	29.07	27.00	33.27	34.74	31.77	34.73
45–49 years	29.50	25.15	32.02	29.86	34.10	29.40	28.14	32.28
50–54 years	28.12	22.30	29.16	30.06	34.09	28.98	28.40	34.83
55–59 years	24.93	16.89	24.00	20.56	28.06	30.88	26.31	30.25
60–64 years	21.28	27.37	26.79	16.84	29.57	25.92	25.80	38.85
65–69 years	16.57	19.14	21.01	28.34	41.00	27.81	32.43	33.21
70–74 years	22.98	25.31	28.45	17.56	41.33	33.84	31.99	52.21
75–79 years	22.97	28.23	38.29	32.31	36.86	29.68	44.34	44.45
**Northeast**								
20–24 years	34.32	27.21	29.09	30.56	32.11	29.61	30.67	35.51
25–29 years	35.53	30.14	29.66	35.73	35.59	32.17	33.03	36.06
30–34 years	31.91	30.62	35.26	35.56	34.56	34.38	33.82	34.83
35–39 years	32.78	28.90	35.27	38.35	37.88	35.88	35.13	35.63
40–44 years	33.33	23.95	33.75	38.85	41.59	37.75	36.61	38.18
45–49 years	34.45	29.09	34.02	38.63	39.58	34.93	38.21	42.91
50–54 years	30.96	29.64	35.24	42.20	38.63	38.43	36.61	43.50
55–59 years	36.74	29.89	32.35	37.11	33.15	37.96	40.21	40.77
60–64 years	36.97	37.38	31.75	38.08	37.57	36.90	38.39	41.03
65–69 years	31.79	26.85	38.55	42.04	31.02	35.60	42.22	49.43
70–74 years	36.67	37.69	37.76	44.90	34.12	36.14	46.52	50.88
75–79 years	44.10	31.75	37.67	42.26	36.65	37.61	40.02	51.47
Central-West								
20–24 years	35.29	24.89	29.99	33.92	32.82	33.17	31.88	40.92
25–29 years	29.49	24.52	31.86	33.48	29.35	30.59	34.52	39.75
30–34 years	29.22	20.74	29.24	35.16	32.42	29.22	36.05	36.66
35–39 years	28.59	25.83	26.26	35.89	29.38	29.02	31.27	39.15
40–44 years	26.13	24.21	27.54	35.95	31.27	33.24	33.9	38.16
45–49 years	23.71	27.04	28.15	37.23	34.96	34.95	29.4	35.43
50–54 years	24.50	19.61	28.25	44.78	34.11	30.46	33.07	32.91
55–59 years	26.48	27.51	31.03	38.19	36.06	32.50	35.57	36.56
60–64 years	23.39	22.78	30.06	46.72	34.73	35.56	32.39	35.89
65–69 years	22.34	23.13	31.71	49.89	35.68	37.50	33.33	41.25
70–74 years	36.75	31.18	33.14	46.34	47.75	33.80	42.13	40.14
75–79 years	32.37	30.67	34.11	58.73	35.47	36.62	39.7	55.52
**South**								
20–24 years	20.74	20.27	23.81	24.72	26.19	25.74	23.82	32.28
25–29 years	20.81	18.65	24.19	23.20	27.98	27.75	27.10	31.12
30–34 years	22.39	19.25	25.55	23.08	26.91	27.85	28.50	32.71
35–39 years	23.34	21.11	25.39	28.65	28.88	29.68	31.15	35.01
40–44 years	27.02	23.31	31.20	29.79	35.42	35.27	35.74	38.65
45–49 years	30.80	33.60	37.71	34.33	39.59	37.60	38.14	42.62
50–54 years	35.42	32.88	38.35	38.16	39.17	36.89	39.75	46.52
55–59 years	37.43	34.98	41.87	38.92	39.95	38.28	41.48	45.84
60–64 years	35.98	35.15	41.10	40.30	40.62	37.51	41.10	45.26
65–69 years	39.43	37.80	45.22	43.56	42.32	41.66	43.50	46.65
70–74 years	47.62	48.13	52.83	43.13	40.97	43.24	50.86	49.87
75–79 years	46.46	49.36	58.06	48.67	52.79	43.54	47.82	54.22
**Southeast**								
20–24 years	27.01	24.16	26.64	29.21	28.44	31.23	32.15	35.22
25–29 years	27.51	26.50	28.47	30.38	31.06	33.62	36.65	38.48
30–34 years	25.80	28.19	28.38	29.66	30.32	34.74	38.40	39.42
35–39 years	24.02	25.62	29.32	32.04	31.47	37.89	40.46	42.27
40–44 years	24.21	24.60	26.15	31.66	33.54	37.69	39.92	41.18
45–49 years	26.05	25.29	28.14	32.12	35.43	38.44	38.99	41.67
50–54 years	25.90	25.33	29.12	30.03	37.66	34.36	37.60	39.40
55–59 years	26.54	29.09	26.78	33.42	37.54	32.79	34.13	38.99
60–64 years	23.94	27.40	24.17	27.59	31.53	32.51	31.48	34.80
65–69 years	27.28	28.95	29.12	27.26	29.25	30.59	31.77	34.14
70–74 years	26.38	36.45	35.21	31.76	33.46	29.30	30.90	33.93
75–79 years	34.81	38.90	35.26	39.15	33.04	28.89	34.43	30.84

One limitation to this modeling is the problem of identifiability. One of the resources used to mitigate this problem is the use of estimable functions in the models. Since the model for the principal effect of age provides a better distinct fit than the models of the principal effect of period and cohort, age was implemented as a mandatory component of preliminary two-factor models, and as a target factor for subsequent restrictions. Preliminary age-period (AP) and age-cohort (AC) models served to develop a complete APC model [25]. Thus, estimable functions are limited to analyzing the linear combinations and curvatures (or deviations from linearity) for age, period, and birth cohort. The curvatures can be estimated and remain constant regardless of the parametrization employed in the analysis. At the same time, the linear combinations are divided into two distinct components, the linear effect of age and the drift effect (corresponding to the linear effect of period and cohort combined). The first drift described in the model represents the linear trend of the logarithm for the age-specific rates. It is equal to the sum of the period and cohort slopes (βL + γL), where βL and γL are the linear period and cohort trends, respectively. In contrast, the second drift represents the longitudinal age trend and is the sum of age and the period slope (*αL + βL*), where *αL* and *βL* are the linear age and period trends, respectively [35, 45, 49].

The model’s fit was performed via deviance, defined as twice the logarithm of the complete model’s likelihood function about the logarithm of the estimated model’s likelihood function [48, 50]. The effects’ contribution was assessed by comparison of the model’s deviance with the specific effect concerning the complete model. Statistical significance of the results was set at P < 0.05 [50]. The model with the lowest deviance has the best fit [48]. The cohort adopted as the reference was 1946, and the reference period was 1998, since they were more centralized in the analysis. The measure of association generated by the APC model is the relative risk (RR) [50], which is calculated automatically by the *apc.fit* function of the Epi package, together with the respective 95% confidence intervals (95%CI) [50].

## Results

From 1981 to 2020, there were 272,716 suicides among individuals between 20 and 79 years of age in Brazil, of which 79.2% were committed by men. The region with the largest share of suicide deaths was the Southeast (40.3%), followed by the South (26.4%), Northeast (19.5%), Central-West (8.4%), and North (5.4%) (data not tabulated).

Suicide mortality rates have shown an upward pattern in the Brazilian population with increasing age and were considerably higher at 70 years and older, with the oldest cohorts showing the highest rates. The age brackets from 50 to 70 years have not shown a specific pattern, while the younger brackets (from 20 to 50 years) have shown an upward pattern in the rates. In the North, suicide mortality rates among younger individuals have increased more than in the other age brackets. The pattern for the age brackets from 20 to 34 years have been upward, while from 35 to 69 years they have declined in the last 5 years. The age brackets from 70 to 74 and from 75 to 79 years showed a distinct pattern from the others, alternating periods of decline with subsequent increases. In Northeast Brazil, most of the rates were upward, except for the 30 to 59 years bracket, which showed a decline at the end of the period. In the Central-West region, the age brackets from 70 years upward have shown higher rates than the others. The rates showed an overall decline in the last 10 years of the follow-up period. With Brazil’s highest rates in the South, the pattern has been downward both for the period and for age brackets. In the Southeast, the cohorts with the highest rates were from 1906 to 1925. The age brackets from 20 to 45 years displayed an upward pattern in recent periods, while the group of individuals 70 and older increased again after a rate decline (Table 1).

Analysis of the likelihood ratio showed that nearly all of the analyses performed in the complete APC model displayed a better fit to the data (p < 0.001) than the other models, except when considering the North, in which the best fit was with the age-drift* model (non-linear effect of age) for the general population. The cohort effect was better in explaining the behavior of suicide rates in the general population, except in the Central-western region. In the male population, this effect was also responsible for the behavior of the Northern, Northeastern and Southeastern regions. In the female population, the period effect explained most of the situations, except for the Central-western and Southern regions (Table 2).

**Table 2:** Fit parameters for APC model of suicide data in Brazil and major geographic regions, 1981 to 2015, in the overall population and by sex

Model	Brazil			North					Northeast			Central-West			South			Southeast		
	df	*RD*	p	df	*RD*	p	Df	*RD*			p	df	*RD*	p	df	*RD*	p	df	*RD*	p
**Overall Population**																				
Age	91	3381.6		91	386.7		91	722.4				91	381.0		91	606.6		91	2560.7	
Age-drift*	90	704.7	< 0.001	90	192.9	< 0.001	90	523.2			< 0.001	90	239.3	< 0.001	90	228.5	< 0.001	90	558.1	< 0.001
Age-Cohort	87	602.7	< 0.001	87	187.9	0.171	87	446.4			< 0.001	87	234.2	0.161	87	165.8	< 0.001	87	461.5	< 0.001
Age-Period-Cohort	84	513.6	< 0.001	84	183.6	0.232	84	403.1			< 0.001	84	186.7	< 0.001	84	130.5	< 0.001	84	457.6	0.271
Age-Period	87	612.7	< 0.001	87	186.8	0.358	87	475.9			< 0.001	87	193.5	0.078	87	194.7	< 0.001	87	554.1	< 0.001
Age-drift**	90	704.7	< 0.001	90	192.9	0.108	90	523.2			< 0.001	90	239.3	< 0.001	90	228.5	< 0.001	90	558.1	0.256
**Male Population**																				
Age	91	3269.1		91	404.8		91	797.1				91	385.4		91	561,6		91	2188.3	
Age-drift*	90	719.3	< 0.001	90	185.0	< 0.001	90	515.9			< 0.001	90	216.8	< 0.001	90	214.7	< 0.001	90	497.1	< 0.001
Age-Cohort	87	691.0	< 0.001	87	178.4	0.087	87	394.0			< 0.001	87	214.8	0.579	87	193.1	< 0.001	87	404.3	< 0.001
Age-Period-Cohort	84	471.5	< 0.001	84	178.0	0.931	84	344.5			< 0.001	84	163.1	< 0.001	84	122.6	< 0.001	84	363.3	< 0.001
Age-Period	87	515.3	< 0.001	87	184.9	0.075	87	454.3			< 0.001	87	168.0	0.174	87	153.3	< 0.001	87	450.0	< 0.001
Age-drift**	90	719.3	< 0.001	90	185.0	0.991	90	515.9			< 0.001	90	216.8	< 0.001	90	214.7	< 0.001	90	497.1	< 0.001
**Female Population**																				
Age	91	743.9		91	154.2		91	291.2				91	125.9		91	223.9		91	750.7	
Age-drift*	90	542.5	< 0.001	90	147.6	0.010	90	281.5			< 0.001	90	119.5	0.011	90	183.6	< 0.001	90	471.3	< 0.001
Age-Cohort	87	442.0	< 0.001	87	140.1	0.056	87	262.4			< 0.001	87	113.8	0.126	87	120.4	< 0.001	87	418.5	< 0.001
Age-Period-Cohort	84	262.5	< 0.001	84	115.9	< 0.001	84	234.8			< 0.001	84	98.8	0.001	84	77.8	< 0.001	84	256.5	< 0.001
Age-Period	87	342.3	< 0.001	87	126.5	0.014	87	252.2			< 0.001	87	104.4	0.132	87	133.3	< 0.001	87	307.3	< 0.001
Age-drift**	90	542.5	< 0.001	90	147.6	< 0.001	90	281.5			< 0.001	90	119.5	0.001	90	183,6	< 0.001	90	471.3	< 0.001

df = degrees of freedom; RD = residual deviance; * linear trend for period and cohort; ** longitudinal trend for age

**Fig. 1:**
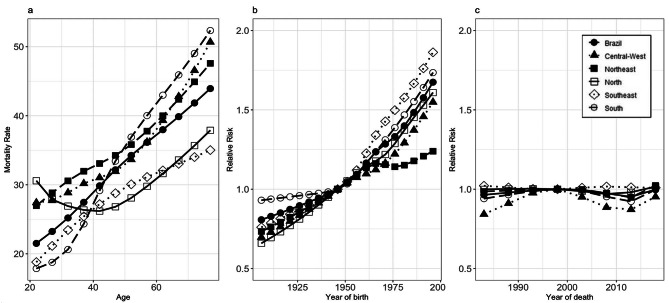
Models adjusted by age, period, and cohort for suicide mortality in the overall population, Brazil and regions, 1981 to 2020. Figure 1a: Age effect. Figure 1b: Period effect. Figure 1c: Cohort effect

In the overall population, the age model-adjusted suicide mortality rates showed an upward pattern (Fig. 1a). The mortality risk ratio (expressed as RR) according to the cohort in the Brazilian population increased starting with the 1946 cohort (reference), and the most recent cohort showed the highest associated risk, 1.67 (95%CI 1.63; 1.71), while the oldest cohort showed the lowest associated risk, 0.80 (95%CI 0.78;0.83). This pattern occurred across all regions: Southeast (1996–2000 cohort, RR = 1.86, 95%CI 1.79;1.93; 1906–1910 cohort, RR = 0.76, 95%CI 0.72;0.80), South (1996–2000 cohort, RR = 1.73, 95%CI 1.62;1.85; 1906–1910 cohort, RR = 0.93, 95%CI 0.85;1.01), North (1996–2000 cohort, RR = 1.60, 95%CI 1.47;1.75; 1906–1910 cohort, RR = 0.65, 95%CI 0.58;0.75), Central-West (1996–2000 cohort, RR = 1.54, 95%CI 1.41;1.69; 1906–1910 cohort, RR = 0.70, 95%CI 0.60;0.80), and Northeast (1996–2000 cohort, RR = 1.23, 95%CI 1.18;1.29; 1906–1910 cohort, RR = 0.73, 95%CI 0.69;0.78)) (Fig. 1b). For the period effect, the Brazilian population showed the highest risk in 1996–2000 (reference, RR = 1.0), the same for the South and Central-West. Northeast and North regions presented higher risks between 2006 and 2010 (RR = 1.02, 95%CI 1.00;1.04 and RR = 1.016; 95%CI 0.98; 1.05, respectively). The Southeast, however, presented a higher risk from 1981 to 1985 (RR 1.02, 95%IC 0.99;1.05) (Fig. 1c).

**Fig. 2:**
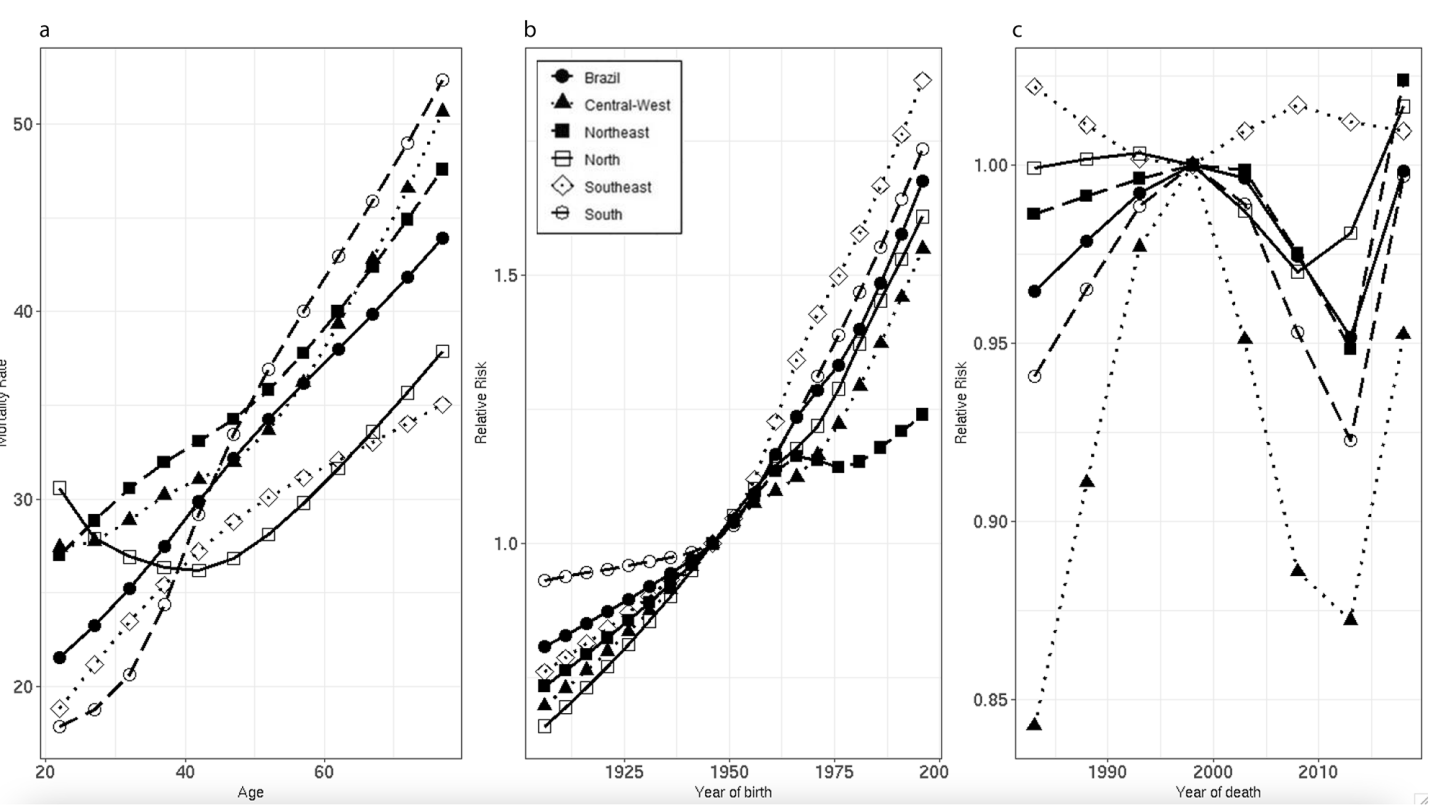
Models adjusted by age, period, and cohort for suicide mortality in the male population, Brazil and regions, 1981 to 2020. Figure 2a: Age effect. Figure 2b: Period effect. Figure 2c: Cohort effect

Considering the male population (the principal victims of death by suicide), the suicide-adjusted mortality rates presented an upward pattern with increasing age (Fig. 2a). As with the overall population, the RR for the male population increased since 1906 cohort (RR = 0.74; 95%CI 0.71;0.77), and the highest risk was in the most recent cohort, RR 1.74 (95%CI 1.69;1.79). This pattern occurred across all regions: Southeast (1996–2000 cohort, RR = 1.90, 95%CI 1.82;1.98; 1906–1910 cohort, RR = 0.75, 95%CI 0.70;0.79), North (1996–2000 cohort, RR = 1.78, 95%CI 1.62;1.96; 1906–1910 cohort, RR = 0.64, 95%CI 0.55;0.73), South (1996–2000 cohort, RR = 1.73, 95%CI 1.62;1.85; 1906–1910 cohort, RR = 0.82, 95%CI 0.75;0.91), Central-West (1996–2000 cohort, RR = 1.70, 95%CI 1.54;1.88; 1906–1910 cohort, RR = 0.63, 95%CI 0.54;0.74), and Northeast (1996–2000 cohort, RR = 1.29, 95%CI 1.23;1.37; 1906–1910 cohort, RR = 0.63, 95%CI 0.58;0.67) (Fig. 2b). As for period effect, the male population presented the highest risk in 2016–2020 in the Northeast region (RR = 1.005; 95%CI 0.98;1.03), and in 2001–2005 in the Southeast region (RR = 1.001; 95%CI 0.99; 1.01). In Brazil, North, South, and Central-West, all the periods showed lower risk than the reference period (Fig. 2c).

**Fig. 3:**
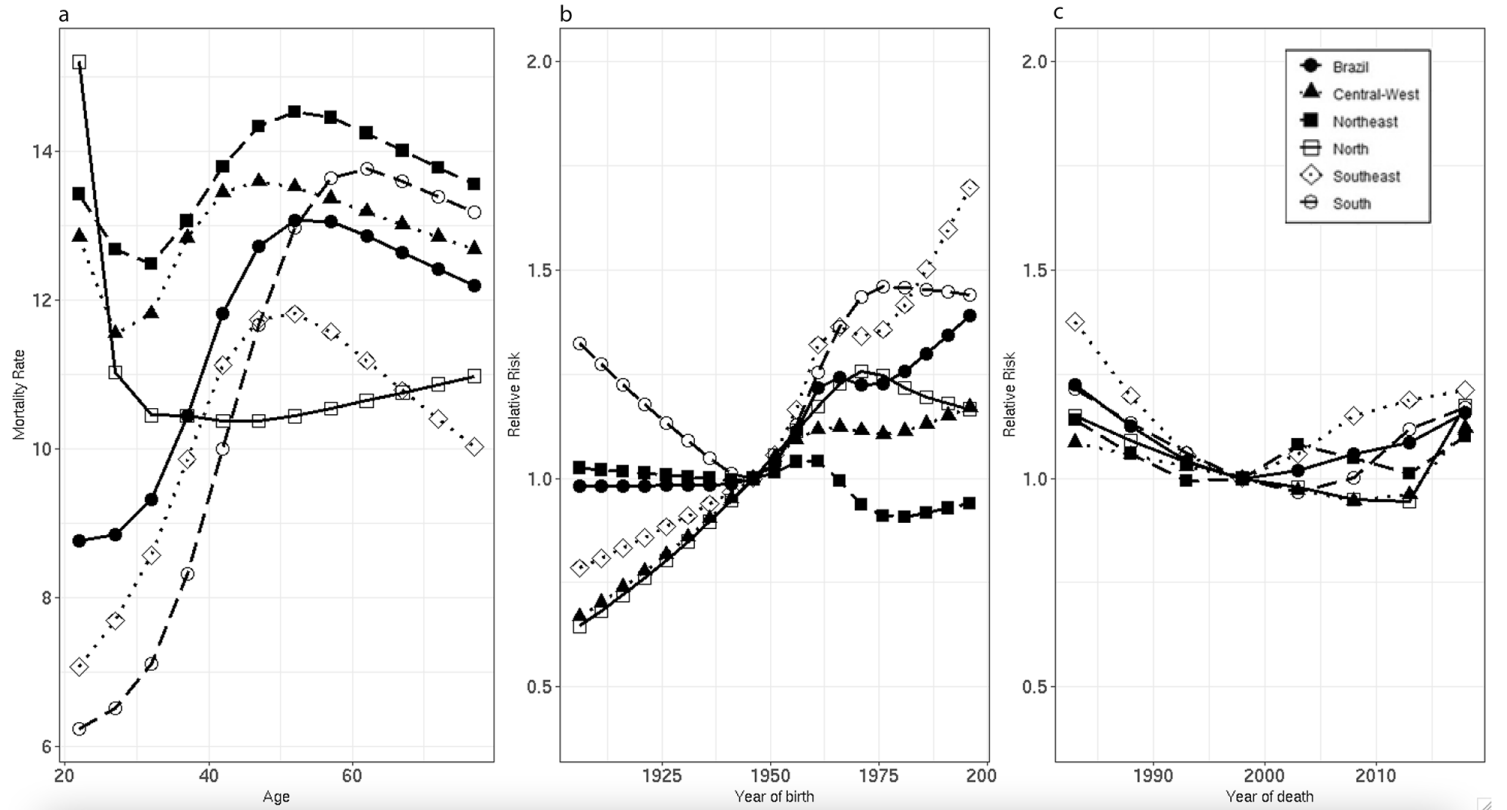
Models adjusted by age, period, and cohort for suicide mortality in the female population, Brazil and regions, 1981 to 2020. Figure 3a: Age effect. Figure 3b: Period effect. Figure 3c: Cohort effect

For the female population in Brazil, the highest rates were between 45 and 60 years of age, decreasing near the end of life. This fact is repeated in the Northeast, Southeast, and Central-West regions. North showed an atypical pattern: the highest rate was from 20 to 24 years, with a decrease in the following age group and a slow increase over the course of the subsequent ages. In South region, rates peaked after 60 years of age (Fig. 3a). The RR for cohort in the Brazilian population was 0.98 (95%CI 0.911;1.05) in the 1906–1910 cohorts followed by increasing risk until the 1996–2000 cohort (RR = 1.39, 95%CI 1.32; 1.46), in the same way as the Central-Western (RR = 1.17; 95%CI 0.97; 1.41) and Southeast Region (RR = 1.69; 95%IC 1.56;1.83). In the North, the risk increased over the course of the cohorts, with a peak in the 1971–1975 cohort (RR = 1.25, 95%CI 1.10;1.43), while the peak was in 1961–1965 (RR = 1.04; 95%CI 0.98;1.10) in the Northeast Region. In the Southern region, all cohorts had a higher risk than the reference cohort (Fig. 3b). As for the period effect, the Brazilian female population had the highest risk in 1981–1985 (RR = 1.22; 95%CI 1.18;1.27), with the same happening in Southeast (RR = 1.37; 95%CI 1.30;1.44), South (RR = 1.21; 95%IC 1.11;1.33), and Northeast (RR = 1.14, 95%CI 1.07;1.22). In the North, and Central-West regions, the period with the highest risk was 2016–2020 (RR = 1.17, 95%CI 1.08;1.28; RR = 1.12; 95%CI 1.03;1.21, respectively) (Fig. 3c).

## Discussion

Our results highlighted how temporal and regional effects had influenced suicide in the Brazilian population. Although for many years Southern Brazil had the highest number of cases and rates [16, 24, 52, 53], it exhibited a pattern of declining rates over the period studied and relative risk in the most recent cohorts. Meanwhile, the Northeast showed growth in suicide rates and the Southeast witnessed an increase in suicide risk in the most recent years analyzed.

Age has always been seen as related to illness and death [36, 54], and is considered a major risk factor for suicide [55, 56], with younger [16, 17, 55, 57–59] and older people [16, 56, 59–64] being more affected. Our study revealed a clear relationship between increasing lifetime suicide rates in virtually all subgroups tested. Only women aged 20–24 in the North had higher suicide rates than all other age groups. Suicide causes in young people are unemployment, economic difficulties, family breakdown, and societal changes (decreased religiousness, new gender roles, and increasing competition in school) [58]. In older people, the problems are different: the impossibility of coping with life as previously, loss of life companions, and disabilities resulting from illnesses or aging itself [65]. In addition, elderly people are also influenced by generational issues.

This study also showed that gender and age were the most important factors for explaining suicide rates in Brazil, as in the other study with APC modeling in Brazil [32] and other countries [25, 27–30]. A major stress factor for men would be the failure to fulfill traditional gender roles, feeling more sensitive to economic setbacks (such as unemployment) [17]. Although women showed a higher propensity for attempts [66–68], they showed more protective factors for completed suicide because they have a lower prevalence of alcoholism, higher religiosity, flexibility in social skills and roles throughout life, recognize risk signs of depression earlier, and participate more in social support networks [69–72].

The generation effect has received little attention. Brazilian society is undergoing an accelerated demographic transition in which personal relations have changed greatly: fertility has decreased, with earlier sexual maturity and viability of pregnancies in older women, as well as procedures that have extended men’s sexual life; changes in the nuclear family; and despite social networks and the modern world’s dynamics. This process of social change with an older population (more physically, economically, and technologically limited) aggravates isolation and depression. Our results show that the most recent cohorts (1991 to 1995) presented a higher suicide risk. The South was the only country region where the oldest cohort (1906 to 1910) also presented an increased risk.

Other studies have largely pointed out these effects on suicide rates. In Switzerland, the cohort effects were similar for males and females, although less pronounced [25]. A study in Spain found a period effect for the female population, while the cohort effect was more evident in the male population [28]. In South Korea, cohort effects were determinant in the rate changes from 1984 to 2013 [30]. A study conducted in Hong Kong and Taiwan found that the age effects for both regions in both sexes were quite similar and suicide rates increased with increasing age. Regarding period effects, Hong Kong had one peak (1999–2003), and Taiwan had two peaks (1979–1983 and 2004–2008). As for cohort effects, in both Hong Kong and Taiwan, younger male cohorts showed high suicide risk; younger female cohorts showed relatively low risk [27]. In the state of Rio de Janeiro, Brazil, from 1979 to 1998, the age-adjusted rates increased, more in men than in women, while a weak period effect was seen in the increasing rates in 1983–1984 and the cohort effect showed a decrease in the rates between the oldest cohorts and the youngest [32]. In our study, the cohort effect was larger than the period effect on suicide rates for most situations.

Period effect summarises a complex set of historical events and environmental factors, such as world wars, economic booms and recessions, famine, infectious disease pandemics, public health interventions, and technological discoveries [26]. Our findings showed that an increase in suicide risk in the period from 2001 to 2005 in the overall population of Brazil and some regions suggested that this period of economic and social transition with changes in the political profile resulted in conditions of emotional instabilities that provided fertile ground for self-destructive behavior. A study in Spain showed that socioeconomic and structural changes were responsible for the increase in depression, alcoholism and suicidal ideation in the 1980s [28]. Likewise, in Switzerland, the two World Wars and economic problems affected suicide rates in both sexes [25]. In Russia, the Cold War, Mikail Gorbatchev’s plans, and the breakup of the Soviet Union were the backdrop for the increase in suicide rates among Russians [29].

Except for the overall population of the North, the model that best fits the data is the complete model (age-period-cohort). Table 1 shows that the cohort effect had a stronger influence than the period effect for the overall and male population. The period effect had a stronger influence on the female population.

The APC method evidenced differences in suicide between males and females. Our findings evidenced that Brazilian women showed an upward pattern in suicide rates throughout adulthood, until around age 50, when rates decreased, except in the Northeast and Southeast regions, where they continued to increase.

### Limitations

Possible limitations are the type of data used (death certificate records) and uncertainty concerning this data’s quality. In addition, the study design (ecological) does not allow individualization of the findings, making it difficult to correlate the suicide event with depression and mental disorders in the Brazilian population and thus address possible causes of this problem.

Regarding the method used here, APC modeling could be more routine and complex to analyze. In other studies that use APC analysis of suicide data, there was no consensus on the best way to analyze the data [27–33]. The current study shares the limitations common to APC analysis. On the one hand, they are constrained by the lack of definitive real-world models, so some ambiguity in the results cannot be ruled out. On the other, the effects of APC represent standard dimensions related to time and age, which are insignificant per se, but provide valuable tools for examining real-world variables. The analysis was also limited to the principal effects and left some room for more complex models, such as non-linear models or models including interaction effects.

Despite these possible limitations, age-period-cohort modeling can describe suicide trends more accurately than other approaches. It does not rule out the need for other epidemiological studies but focuses on where it has proven most urgent and where the interventions should begin.

## Conclusions

Suicide mortality rates have shown an upward pattern with increasing age in the Brazilian population, independently of gender. However, the behavior of period and cohort effects depends on the population analyzed and its regional distribution. According to our findings, many of Brazil’s regions were more influenced by a period effect, and fluctuations in the patterns were consistent with periods of economic growth versus recession. This suggests a guideline that can be followed to promote an effective public policy in mental health.

Today, suicide prevention in Brazil is still a low-priority public policy. “Yellow September” has been celebrated in recent years as part of the prevention strategy, but precarious mental healthcare at the local level (in Brazil’s municipalities) is still an obstacle to accessing this type of service. More services and more healthcare professionals are needed to treat all aspects of psychological distress, ranging from primary care to Centers for Psychosocial Support and referral hospitals for psychiatric care. It is important to eliminate deeply rooted taboos and prejudices and to understand that the process of suffering that leads from ideation to death by suicide can be interrupted, thus avoiding early loss of lives and sequelae from suicide attempts.

## Data Availability

The datasets analysed during the current study are available in the Zenodo repository, https://zenodo.org/record/6547039#.YoJ-QOjMK3C.

## References

[1] Barbosa F, de O, Macedo PCM, Silveira RMC. da. Depressão e o suícido. Rev SBPH. 2011;14:233–43.

[2] Ribeiro NM, Castro S, de Scatena S, Haas LM (2018). Análise da tendência temporal do suicídio e de sistemas de informações em saúde em relação às tentativas de suicídio. Texto Contexto - Enferm.

[3] de Sousa GS, Silva RM da, Figueiredo AEB, Minayo MC, de Vieira S. S. Circunstâncias que envolvem o suicídio de pessoas idosas. Interface - Comun Saúde Educ. 2014;18:389–402.

[4] Brasil O. Pan-Americana da Saúde. Painel de Indicadores do SUS, 5.

[5] WHO. Suicide. World Health Organization. 2021. https://www.who.int/news-room/fact-sheets/detail/suicide. Accessed 31 Oct 2021.

[6] Dantas ESO. Prevenção do suicídio no Brasil: como estamos? Physis Rev Saúde Coletiva. 2019;29.

[7] Associação Brasileira de Psiquiatria (ABP) (2014). Suícidio: informando para prevenir.

[8] de Souza ER, Minayo MC, de Malaquias S (2002). Suicide among young people in selected brazilian state capitals. Cad Saúde Pública.

[9] Machado DB, dos Santos DN (2015). Suicídio no Brasil, de 2000 a 2012. J Bras Psiquiatr.

[10] Vasconcelos AMN, Gomes MMF (2012). Transição demográfica: a experiência brasileira. Epidemiol E Serviços Saúde.

[11] Macente LB, Zandonade E (2012). Spatial distribution of suicide incidence rates in municipalities in the state of Espírito Santo (Brazil), 2003–2007: spatial analysis to identify risk areas. Braz J Psychiatry.

[12] Parente A, CM, Soares R, de Araújo B, Cavalcante ARF, de Monteiro IS (2007). Caracterização dos casos de suicídio em uma capital do Nordeste Brasileiro. Rev Bras Enferm.

[13] Brazil (2021). Mortalidade por suicídio e notificações de lesões autoprovocadas no Brasil. Bol Epidemiológico.

[14] Brazil (2017). Perfil epidemiológico das tentativas e óbitos por suicídio no Brasil e a rede de atenção à saúde. Bol Epidemiológico.

[15] da Mata KCR, Daltro MR, Ponde MP (2020). Perfil epidemiológico de mortalidade por suicídio no Brasil entre 2006 e 2015. Rev Psicol Divers E Saúde.

[16] de Mello-Santos C, Bertolote JM, Wang Y-P (2005). Epidemiology of suicide in Brazil (1980–2000): characterization of age and gender rates of suicide. Braz J Psychiatry.

[17] Meneghel SN, Victora CG, Faria NMX, de Carvalho LA, Falk JW (2004). Características epidemiológicas do suicídio no Rio Grande do sul. Rev Saúde Pública.

[18] Nock MK, Borges G, Bromet EJ, Cha CB, Kessler RC, Lee S (2008). Suicide and suicidal behavior. Epidemiol Rev.

[19] Vasconcelos-Raposo J, Soares AR, Silva F, Fernandes MG, Teixeira CM (2016). Níveis de ideação suicida em jovens adultos. Estud Psicol Camp.

[20] Reichenheim ME, de Souza ER, Moraes CL, Jorge MHP, de Silva M. CMFP da, Minayo MC de S. Violence and injuries in Brazil: the effect, progress made, and challenges ahead. The Lancet. 2011;377:1962–75. 10.1016/S0140-6736(11)60053-6 21561649

[21] Silva TL, Maranhão TA, Sousa GJB, Silva IG, da, Lira Neto JCG (2022). Araujo GA dos S. Análise espacial do suicídio no nordeste do Brasil e fatores sociais associados. Texto Contexto - Enferm.

[22] Gonçalves AM, Freitas PP (2011). Sequeira CA da C. Comportamentos suicidários em estudantes do ensino superior: factores de risco e de protecção. Millenium.

[23] Cardoso HF, Baptista MN, Ventura CD, Branão EM, Padovan FD, Gomes MA (2012). Suicídio no Brasil e América Latina: revisão bibliométrica na base de dados redalycs. Diaphora.

[24] Lovisi GM, Santos SA, Legay L, Abelha L, Valencia E (2009). Análise epidemiológica do suicídio no Brasil entre 1980 e 2006. Braz J Psychiatry.

[25] Ajdacic-Gross V, Bopp M, Gostynski M, Lauber C, Gutzwiller F, Rössler W (2006). Age-period-cohort analysis of swiss suicide data, 1881–2000. Eur Arch Psychiatry Clin Neurosci.

[26] Yang Y, Land KC (2013). Age-period-cohort analysis: new models, methods, and empirical applications.

[27] Chen Y-Y, Yang C-T, Pinkney E, Yip PSF (2021). The age-period-cohort trends of suicide in Hong Kong and Taiwan, 1979–2018. J Affect Disord.

[28] Granizo JJ, Guallar E, Rodríguez-Artalejo F (1996). Age-period-cohort analysis of suicide mortality rates in Spain, 1959–1991. Int J Epidemiol.

[29] Jukkala T, Stickley A, Mäkinen IH, Baburin A, Sparén P (2017). Age, period and cohort effects on suicide mortality in Russia, 1956 – 2005. BMC Public Health.

[30] Park C, Jee YH, Jung KJ (2016). Age–period–cohort analysis of the suicide rate in Korea. J Affect Disord.

[31] Phillips JA (2014). A changing epidemiology of suicide? The influence of birth cohorts on suicide rates in the United States. Soc Sci Med 1982.

[32] Rodrigues NCP, Werneck GL (2005). Age-period-cohort analysis of suicide rates in Rio de Janeiro, Brazil, 1979–1998. Soc Psychiatry Psychiatr.

[33] Surtees PG, Duffy JC (1989). Suicide in England and Wales 1946–1985: an age-period-cohort analysis. Acta Psychiatr Scand.

[34] Carstensen B (2005). Demography and epidemiology: age-period-cohort models in the computer age.

[35] Meira KC, Silva GA, da e, Silva CMFP, Valente JG (2013). Efeito idade-período-coorte na mortalidade por câncer do colo uterino. Rev Saúde Pública.

[36] Yang Y, Schulhofer-Wohl S, Fu WJ, Land KC (2008). The intrinsic estimator for Age‐Period‐Cohort Analysis: what it is and how to use it. Am J Sociol.

[37] Rutherford MJ, Lambert PC, Thompson JR (2010). Age–period–cohort modeling. Stata J.

[38] Cuddy-Casey M, Orvaschel H (1997). Children’s understanding of death in relation to child suicidality and homicidality. Clin Psychol Rev.

[39] Bridge JA, Goldstein TR, Brent DA (2006). Adolescent suicide and suicidal behavior. J Child Psychol Psychiatry.

[40] Tishler CL, Reiss NS, Rhodes AR (2007). Suicidal behavior in children younger than twelve: a diagnostic challenge for emergency department personnel. Acad Emerg Med Off J Soc Acad Emerg Med.

[41] Meira KC, Jomar RT, Santos J, dos, Silva GW dos, Dantas S, Resende ESO et al. EB,. Efeitos temporais das estimativas de mortalidade corrigidas de homicídios femininos na Região Nordeste do Brasil. Cad Saúde Pública. 2021;37:e00238319. 10.1590/0102-311X00238319 33624695

[42] Queiroz BL, Freire FHM, de Gonzaga A, de Lima MR (2017). Estimativas do grau de cobertura e da mortalidade adulta (45q15) para as unidades da federação no Brasil entre 1980 e 2010. Rev Bras Epidemiol.

[43] de Lima EEC, Queiroz BL (2014). Evolution of the deaths registry system in Brazil: associations with changes in the mortality profile, under-registration of death counts, and ill-defined causes of death. Cad Saúde Pública.

[44] de Silva LG. C e. Projeções dos níveis e padrões da mortalidade no Brasil e grandes regiões 1950-2010-2110 pelo método coerente Lee-Carter estendido e outros: a tábua BR-geracional e o risco de longevidade nas instituições previdenciárias do país. Tese (Doutorado em Demografia). Universidade Federal de Minas Gerais; 2019.

[45] Holford TR (1991). Understanding the effects of age, period, and cohort on incidence and mortality rates. Annu Rev Public Health.

[46] Yang Y, Land KC (2013). Age-period-cohort analysis: new models, methods, and empirical applications.

[47] Ahmad OB, Boschi-Pinto C, Lopez AD, Murray CJ, Lozano R, Inoue M (2001). Age standardization of Rates: a new WHO Standard. GPE Discuss Pap Ser.

[48] González JR, Llorca FJ, Moreno V (2002). Algunos aspectos metodológicos sobre los modelos edad-período-cohorte: aplicación a las tendencias de mortalidad por cáncer. Gac Sanit.

[49] Holford TR (1983). The estimation of age, period and cohort effects for vital rates. Biometrics.

[50] Meira KC, Guimarães RM, dos Santos J, Cabrelli R (2015). Análise de efeito idade-período-coorte na mortalidade por câncer de mama no Brasil e regiões. Rev Panam Salud Publica.

[51] Robertson C, Boyle P (1998). Age-period-cohort analysis of chronic disease rates. I: modelling approach. Stat Med.

[52] Calixto Filho M, Zerbini T (2016). Epidemiologia do suicídio no Brasil entre os anos de 2000 e 2010. Saúde Ética Justiça.

[53] Viana GN, Zenkner F, de Sakae M, Escobar TM (2008). Prevalência de suicídio no sul do Brasil, 2001–2005. J Bras Psiquiatr.

[54] Robertson C, Gandini S, Boyle P (1999). Age-period-cohort models: a comparative study of available methodologies. J Clin Epidemiol.

[55] Abasse MLF, de Oliveira RC, Silva TC, Souza ER. de. Análise epidemiológica da morbimortalidade por suicídio entre adolescentes em Minas Gerais, Brasil. Ciênc Saúde Coletiva. 2009;14:407–16. 10.1590/s1413-81232009000200010 19197416

[56] Carmo ÉA, Santos PHS, Ribeiro BS, Soares C, de Santana J, Bomfim MLAD dos. S, Características sociodemográficas e série temporal da mortalidade por suicídio em idosos no estado da Bahia, 1996–2013. Epidemiol E Serviços Saúde. 2018;27:e20171971. 10.5123/S1679-49742018000100001 29412350

[57] Cantor CH, Neulinger K (1999). Australian suicide trends 1964–1997: youth and beyond?. Med J Aust.

[58] Mittendorfer-Rutz E (2006). Trends of youth suicide in Europe during the 1980s and 1990s – gender differences and implications for prevention. J Mens Health Gend.

[59] Vidal-Rodeiro CL, Santiago-Pérez MI, Paz-Esquete J, López-Vizcaíno ME, Cerdeira-Caramés S, Hervada-Vidal X (2001). Distribución geográfica y temporal del suicidio en Galicia (1976–1998). Gac Sanit.

[60] Byard RW, Hanson KA, Gilbert JD (2004). Suicide methods in the elderly in South Australia 1981–2000. J Clin Forensic Med.

[61] Cheong K-S, Choi M-H, Cho B-M, Yoon T-H, Kim C-H, Kim Y-M (2012). Suicide rate differences by sex, age, and urbanicity, and related regional factors in Korea. J Prev Med Pub Health.

[62] Kim M-H, Jung-Choi K, Jun H-J, Kawachi I (2010). Socioeconomic inequalities in suicidal ideation, parasuicides, and completed suicides in South Korea. Soc Sci Med 1982.

[63] Minayo MC, de Pinto S, Assis LW, de Cavalcante SG (2012). Mangas RM do N. Tendência da mortalidade por suicídio na população brasileira e idosa, 1980–2006. Rev Saúde Pública.

[64] Pavia M, Nicotera G, Scaramuzza G, Angelillo IF (2005). Suicide mortality in Southern Italy: 1998–2002. Psychiatry Res.

[65] Kjølseth I, Ekeberg O, Steihaug S (2010). Why suicide? Elderly people who committed suicide and their experience of life in the period before their death. Int Psychogeriatr.

[66] Bernardes SS, Turini CA, Matsuo T (2010). Perfil das tentativas de suicídio por sobredose intencional de medicamentos atendidas por um Centro de Controle de Intoxicações do Paraná, Brasil. Cad Saúde Pública.

[67] Ghafarian Shirazi HR, Hosseini M, Zoladl M, Malekzadeh M, Momeninejad M, Noorian K (2012). Suicide in the Islamic Republic of Iran: an integrated analysis from 1981 to 2007. East Mediterr Health J.

[68] Silva LF (1999). Saúde das mulheres: o género, determinante cultural de saúde. Arq Med.

[69] Andrés AR (2005). Income inequality, unemployment, and suicide: a panel data analysis of 15 european countries. Appl Econ.

[70] Koo J, Cox WM (2008). An economic interpretation of suicide cycles in Japan. Contemp Econ Policy.

[71] Rodriguez A. Inequality and Suicide Mortality: A Cross-Country Study. Working Paper. Development Research Working Paper Series; 2006.

[72] Stack S (2000). Suicide: a 15-year review of the sociological literature. Part II: modernization and social integration perspectives. Suicide Life Threat Behav.

